# Improving grape fruit quality through soil conditioner: Insights from RNA-seq analysis of Cabernet Sauvignon roots

**DOI:** 10.1515/biol-2022-0864

**Published:** 2024-05-07

**Authors:** Peng Jiang, Xiaojing Wang, Rui Wang

**Affiliations:** College of Agronomy, Ningxia University, Yinchuan 750021, P.R. China; Ningxia Research Institute of Quality Standards and Testing Technology of Agricultural Products, Yinchuan 750001, P.R. China; Ningxia Grape and Wine Research Institute, Yinchuan 750021, P.R. China

**Keywords:** grapes, fertilizer, soil conditioner, fruit quality, RNA-Seq

## Abstract

The application of fertilizers and soil quality are crucial for grape fruit quality. However, the molecular data linking different fertilizer (or soil conditioner [SC]) treatments with grape fruit quality is still lacking. In this study, we investigated three soil treatments, namely inorganic fertilizer (NPK, 343.5 kg/hm^2^ urea [N ≥ 46%]; 166.5 kg/hm^2^ P_2_O_5_ [P_2_O_5_ ≥ 64%]; 318 kg/hm^2^ K_2_O [K_2_O ≥ 50%]), organic fertilizer (Org, 9 t/hm^2^ [organic matter content ≥ 35%, N + P_2_O_5_ + K_2_O ≥ 13%]), and SC (SC, 3 t/hm^2^ [humic acid ≥ 38.5%; C, 56.1%; H, 3.7%; N, 1.5%; O, 38%; S, 0.6%]), on 4-year-old Cabernet Sauvignon grapevines. Compared with the NPK- and Org-treated groups, the SC significantly improved the levels of soluble solids, tannins, anthocyanins, and total phenols in the grape berries, which are important biochemical indicators that affect wine quality. Furthermore, we conducted RNA-seq analysis on the grapevine roots from each of the three treatments and used weighted gene co-expression network analysis to identify five hub genes that were associated with the biochemical indicators of the grape berries. Furthermore, we validated the expression levels of three hub genes (*ERF*, *JP*, and *SF3B*) and five selected genes related to anthocyanin biosynthesis (*UFGT1*, *UFGT2*, *UFGT3*, *GST*, and *AT*) by using quantitative reverse transcription-polymerase chain reaction. Compared to the NPK and Org treatment groups, the SC treatment resulted in a significant increase in the transcription levels of three hub genes as well as *VvUFGT1*, *VvUFGT3*, *VvGST*, and *VvAT*. These results suggest that the SC can improve grape fruit quality by altering gene transcription patterns in grapevine roots and further influence the biochemical indices of grape fruits, particularly anthocyanin content. This study reveals that the application of SC can serve as an important measure for enhancing vineyard SC and elevating grape quality.

## 1 Introduction

Grape (*Vitis vinifera* L.) is a widely cultivated and economically significant crop worldwide. Owing to its elevated nutritional value, exceptional flavor profile, and substantial profitability, grapes have garnered unprecedented popularity [1,2]. Wine, being the most paramount product of grape processing, exhibits certain effects in anti-aging [3], cerebrovascular protection [4,5], anti-cancerous [6], and so on. As the realization of wine’s significance grew, its demand in China has experienced a rapid increase, propelling China to the helm of the fastest-growing wine consumer nation globally [7]. Currently, emphasis is being placed on wine quality, thereby rendering the improvement of grape quality a topic of great scholarly interest in the domain of grape cultivation research.

The composition of grapes, such as soluble solids, phenolic compounds, tannins, titratable acids, and sugar to acid ratios, largely determines the quality, texture, and aroma of wine [1]. The judicious application of fertilizers can considerably impact the composition and flavor compound of wine grape fruits. Foliar application of different types of nitrogen can have different effects on the formation of mature fruit components, such as ammonium sulfate can significantly increase the content of soluble solids, anthocyanins, and total phenols;, phenylalanine can significantly increase the content of titratable acid and tannins in grape fruits; and urea can increase the contents of total anthocyanins, flavanols, and flavonol in grape skins [8]. Compared with conventional fertilization, foliar spraying with iron (ferrous sulfate, ferric ethylenediaminetetraacetic acid, ferric citrate, ferric gluconate, and ferric sugar alcohol) increased berry sugar content and reduced acid content [9]. In addition, preharvest applications of phenolic acids, such as benzoic acid, oxalic acid, and citric acid (CA), had significant effects on the quality properties of the grapes [10]. These studies indicate that the use of fertilizers can control the biosynthesis of compounds that affect the quality of grape fruit.

However, the excessive and continuous application of traditional chemical fertilizers, especially nitrogen fertilizers, causes soil compaction, decreases soil fertility, accelerates soil acidification, increases the likelihood of pest infestations, and leads to a reduction in organic matter load, humus load, and beneficial organisms [11,12]. Reducing the reliance on conventional chemical fertilizers, increasing the utilization of organic fertilizers, and improving soil quality have become increasingly popular approaches for grape cultivation. Increasing organic fertilizer usage in grape management can significantly improve soil nutrient levels and microbial community composition [13,14], as well as increase the soluble solid content of grape berries, reduce the pH value of grape juice, and enhance the diversity of fungi and the relative abundance of beneficial fungi on grape berry surfaces [15]. In addition, soil conditioners (SC), such as vermicompost, have been shown to effectively reduce copper phytotoxicity in young grapevines grown in soils with high copper contents, as well as enhance the utilization of certain important micronutrients [16]. Cataldo et al. demonstrated that soil amendments can significantly improve the efficiency of grapevines in utilizing soil nutrients and water, reduce their fertilizer requirements, and, most importantly, improve the quality of the grapes [17]. However, these previous studies have focused on the effects of different types of fertilizers or SCs on the related biochemical parameters that affect grape quality, lacking research on the molecular mechanisms of grape under different treatments.

In the present study, Cabernet Sauvignon, a wine grape variety widely cultivated in the foothills of Helan Mountain in China, was used as the plant material. Using RNA-seq technology, we aimed to explore the effects of different soil management measures (conventional inorganic fertilizer, organic fertilizer, and SC) on grapevine root gene expression and to clarify the relationship between molecular effects and grape quality traits. The findings will establish a vital molecular basis for enhancing grape quality and guide future soil management strategies in vineyards.

## 2 Materials and methods

### 2.1 Plant materials and treatments

In this experiment, 4-year-old Cabernet Sauvignon grapevines were cultivated in 2021 at Lilan Chateau (105°58′20ʺ E, 38°16′38ʺ N) located in Ningxia, China. The vineyard is situated at an elevation of approximately 1,160 m and experiences an average annual temperature of 8.5°C. The vineyard soil was classified as sandy loam. The planting direction of the plants was north–south, with an inclined upper frame shape. The plant row spacing was 0.6 m × 3.5 m, and drip irrigation was used as the irrigation method.

Three treatments were set up in this experiment, including inorganic fertilizer (NPK), organic fertilizer (Org), and SC (specific components and doses are shown in Table S1). Each treatment consisted of three replicates, and each replicate included at least 20 grapevines. Fertilization and SC were applied by backfilling the excavated pits of 40 cm once a year prior to plant cultivation. Other management measures, such as irrigation, pruning, and pest control, were kept consistent.

### 2.2 Determination of grape fruit quality

As previously described [8], soluble solids were measured by using a handheld sugar meter, titratable acid content was determined by using a standard 0.1 mol L^−1^ NaOH method, soluble sugars were measured by the anthrone reagent method, and total phenol was measured via the Foling-Shocka method. The pH differential method was used to test anthocyanins, while the Flynn-Dennis method was used to evaluate tannins.

### 2.3 RNA extraction and sequencing

Total RNA was extracted by TRIzol reagent (Invitrogen, Thermo Fisher Scientific Inc., USA) from each root tissue, followed by DNase I digestion (Takara, Dalian, China) as per the manufacturer’s instructions. Subsequently, the integrity of RNAs was determined by checking the ratio of optical density at 260 nm to that at 280 nm (OD260/280 = 1.8–2.0) using an ultraviolet spectrophotometer (Hoefer, MA, USA), as well as visually assessing the integrity of 18s and 28s ribosomal RNAs by electrophoresis in an agarose gel. Agilent 2100 Bioanalyzer (Agilent Technologies, Palo Alto, CA, USA) and Qubit 2.0 Fluorometer (Agilent Technologies) were used for the determination of library quality. The Illumina Hiseq pair-end 150 platform was employed for the sequencing.

### 2.4 Data processing and gene expression analysis

Downloaded sequencing data (FASTQ files) were proceeded using FastQC (v1.11.5; http://www.bioinformatics.babraham.ac.uk/projects/fastqc/). Clean data were then extracted after removing low-quality reads and adapters. The ribosome RNA sequences with >5 mismatch bases were removed. The mapping of clean reads to reference *V. vinifera* genome was performed using TopHat2 software [18]. Cufflinks software was used to calculate gene FPKM values (mean fragments per kilobase of transcript per million mapped reads) [19]. Novel genes (with length ≥200 bp and exon number ≥2) were annotated against the Kyoto Encyclopedia of Genes and Genomes (KEGG) and gene ontology (GO). Principal component analysis (PCA) and Pearson’s correlation analysis were performed based on the FPKM value of each gene. Differentially expressed genes (DEGs) between groups were calculated with the criteria of false discovery rate *p* value <0.05.

### 2.5 Construction of gene co-expression network modules

As described by Langfelder and Horvath [20], a weighted gene co-expression network analysis (WGCNA) was introduced in this study to simplify genes into co-expression modules. Specifically, the FPKM values were normalized to create an adjacency matrix, and then the phenotype data were inputted into the WGCNA package to compute the correlation-based associations between gene modules and phenotypes. Within WGCNA, the adjacency matrix was transformed into a topology overlap matrix. Based on the network construction, transcripts exhibiting similar expression patterns were grouped into co-expression modules, and unique genes from these modules were identified. Genes were exported into Cytoscape 3.7.1 using the default parameters from all modules [21].

### 2.6 Quantitative reverse transcription-polymerase chain reaction (qRT-PCR) validation for transcriptome data

Based on the key modules of WGCNA analysis, the gene network was constructed and five hub genes were selected for qRT-PCR. Primers were designed by using Genscript Primer Design online tool (https://www.genscript.com.cn/tools/real-time-pcr-taqman-primer-design-tool). The cDNA was synthesized and subjected to qRT-PCR using gene-specific primers and ChamQ Universal SYBR qPCR Master Mix (Q711-02, Vazyme, Nanjing, China). *Vvactin* was used as a reference gene [22] (all the primers are listed in Supplement data 1: Table S4). The qRT-PCR was conducted with three biological and technical replicates. The relative expression levels were determined by applying the 2^−ΔΔCT^ method.

### 2.7 Data analysis

Three biological replicates were set up throughout the study. We used SPSS for Windows (Chicago, IL, USA) for statistical analyses. The averages (±SE) were submitted for analysis of variance with the Tukey–Kramer test.

## 3 Results

### 3.1 Effect of different treatments on grape berry quality

**Figure 1:**
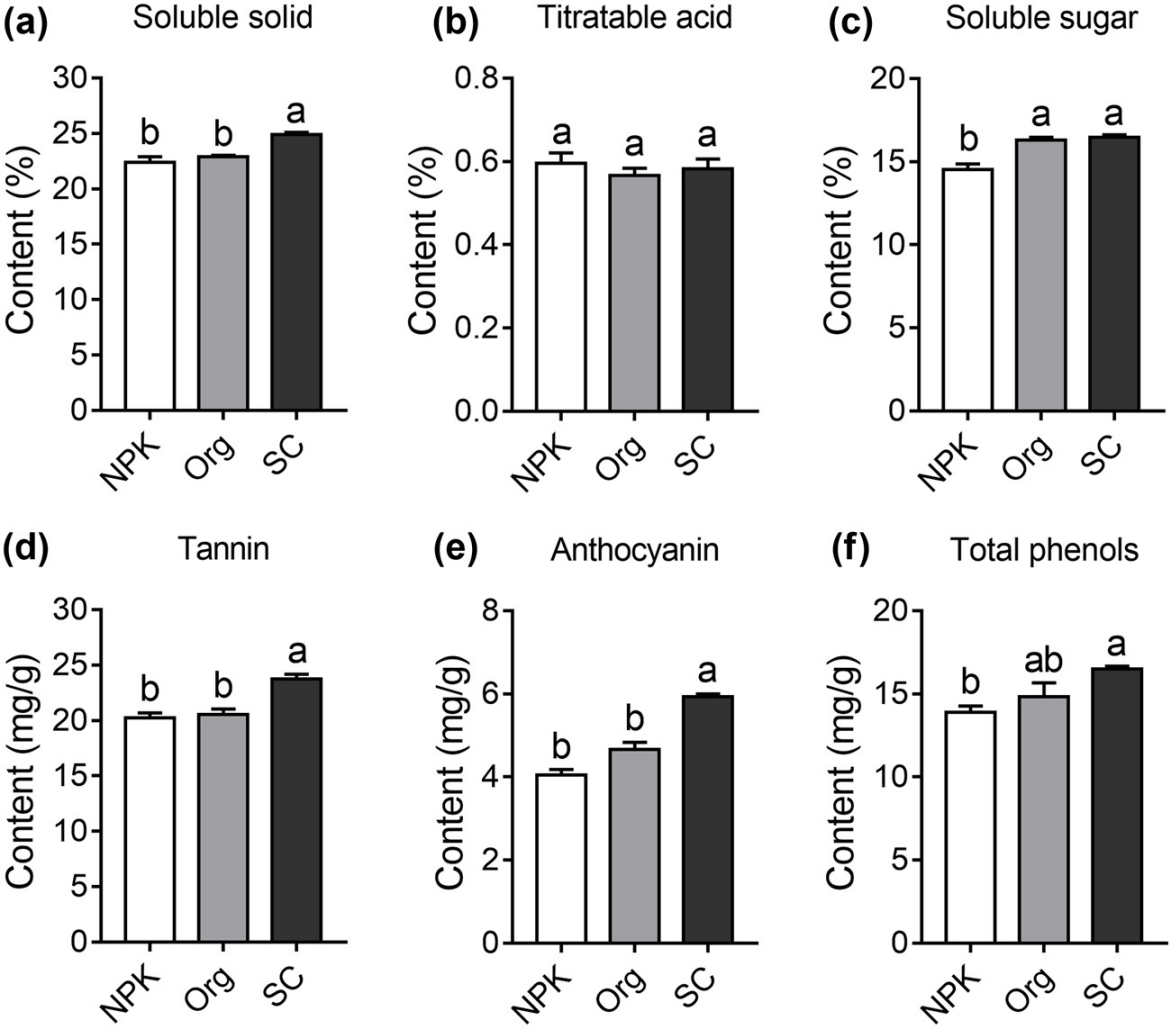
Effect of different treatments on grape berry quality indicators. The levels of soluble solid (a), titratable acid (b), soluble sugar (c), tannins (d), anthocyanins (e), and total phenols (f) in grape berries were measured. The average values (±SE) followed by different letters indicate significant difference at *p* value <0.05 using analysis of variance with the Tukey–Kramer test.

Compared to NPK and Org treatments, SC significantly improved grape fruit quality. Here, six quality indicators of grape fruit were investigated, including soluble solids, titratable acid, soluble sugars, tannins, anthocyanins, and total phenols. Specifically, the average content of soluble solids in the NPK-, Org-, and SC-treated group was 22.4, 22.9, and 24.9%, respectively, with the SC-treated group significantly higher than the other two fertilizer treatments (Figure 1a). There were no significant differences in the titratable acidity levels among the three treatments (Figure 1b). Regarding the soluble sugar content, the SC treatment exhibited a significant increase of 13.4% compared to the NPK treatment, while no significant difference was detected between the SC and NPK treatments (Figure 1c). Moreover, the tannin and anthocyanin levels in grape fruits treated with SC were significantly higher compared to those treated with NPK or Org (Figure 1d and e). The total phenolic level of fruits treated with SC was significantly higher than that of the NPK treatment group, but no significant difference was observed compared to the Org treatment group (Figure 1f).

### 3.2 RNA-seq profiles

To gain a comprehensive understanding of the differences in gene expression within grapevine roots, nine RNA-seq libraries (NPK1, NPK2, NPK3, Org1, Org2, Org3, SC1, SC2, and SC3) were generated. Each library yielded over 45 million clean reads, with more than 70% of these reads mapping to either unique or multiple genomic locations (Table S2).

**Figure 2:**
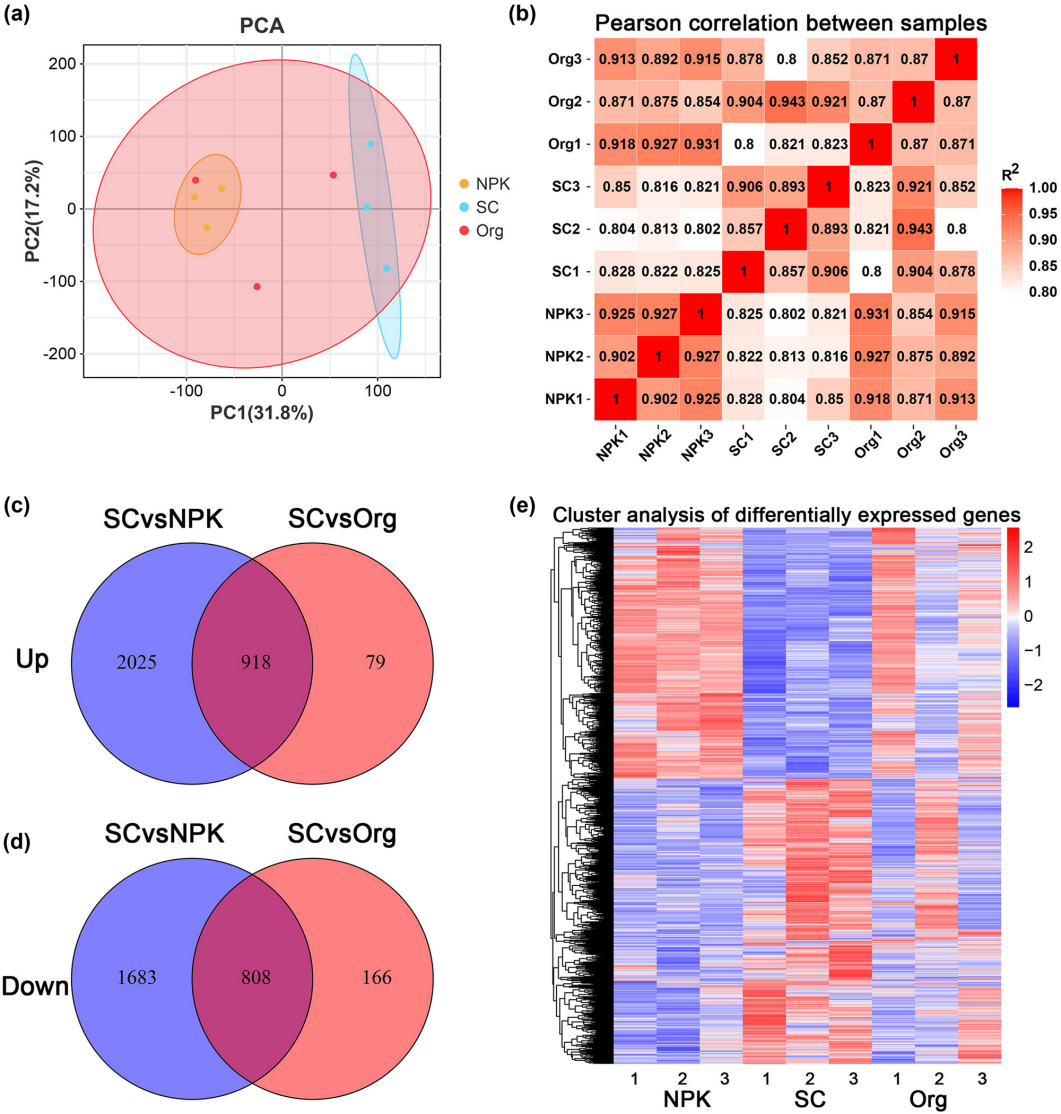
DEG in *V. vinifera* roots after different treatments. (a) PCA analysis of RNA-Seq expression profiles in different treatment conditions. (b) Correlation of RNA-Seq expression profiles of samples in different conditions. Venn diagram of up-regulated genes (c) and down-regulated genes (d) identified under different conditions. (e) Cluster analysis of DEGs in different treatment conditions.

Genes with an average abundance and variance of at least five FPKM were selected to undergo PCA and hierarchical clustering. The first and second principal components accounted for 31.8 and 17.2% of the total variation, respectively (Figure 2a). Pearson correlation analysis revealed strong correlations among the samples between pairwise treatments (Figure 2b).

To investigate the molecular mechanisms underlying the differences between SC and the other two fertilizer treatments, we used the DESeq2 “v1.14.1” package to identify DEGs in different treatments [23]. The selection criteria for DEGs were based on previous study [24]. Compared to the NPK and Org treatment groups, the SC treatment group had 2,943 and 997 up-regulated DEGs, respectively, with 918 overlapping DEGs (Figure 2c). In addition, compared to the NPK and Org treatment groups, the SC treatment group had 2,491 and 974 down-regulated DEGs, respectively, with 808 overlapping DEGs (Figure 2d). Cluster analysis revealed highly similar gene expression profiles among the different treatments, especially within the NPK or SC treatment groups (Figure 2e).

### 3.3 Functional analysis of DEGs

**Figure 3:**
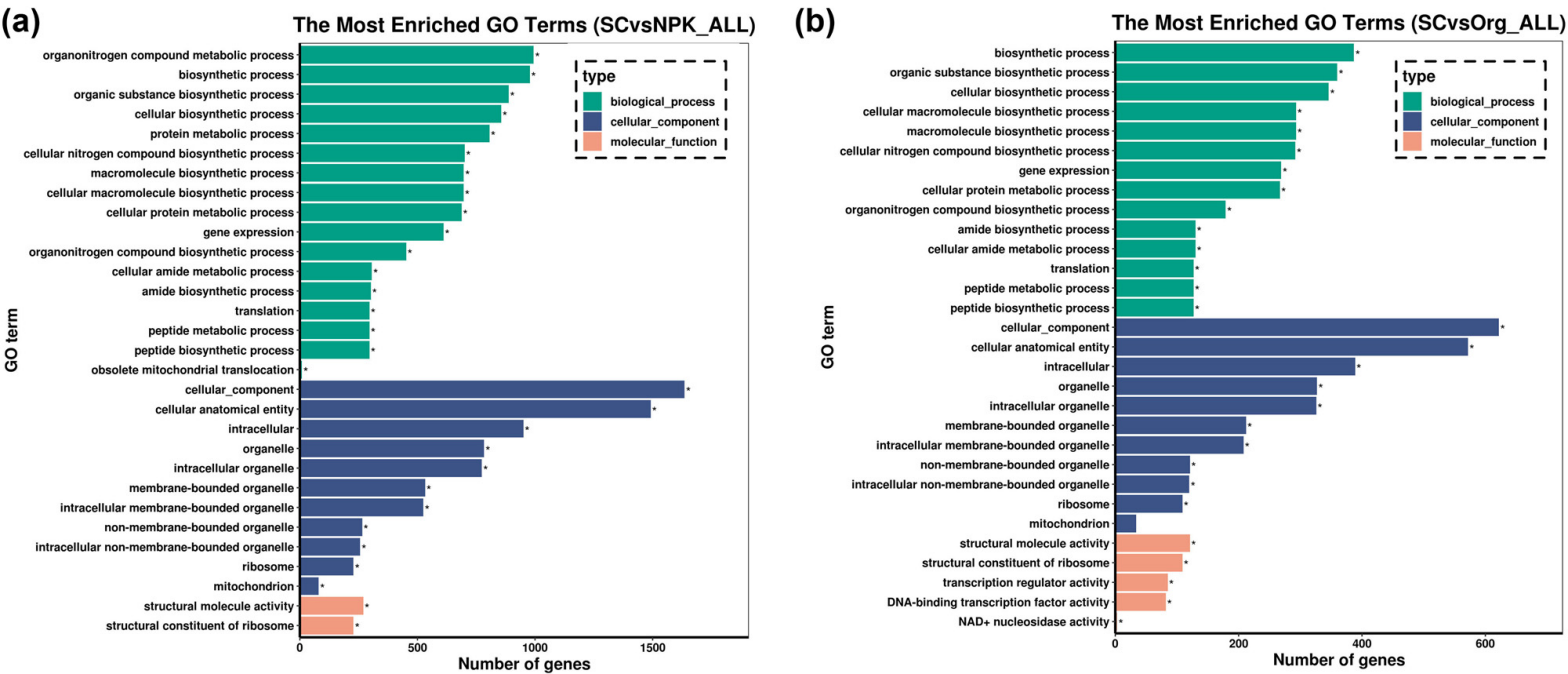
GO enrichment analysis of DEGs: (a) SC vs NPK and (b) SC vs Org.

To analyze the response of genes with different expression levels to different soil treatments, GO was used to enrich for DEGs. As shown in Figure 3, the top 30 GO enrichment entries were categorized into three types: “biological process,” “cellular component,” and “molecular function.” In the results of comparing the SC and NPK treatments (SC vs NPK), the biological process category of GO terms included organonitrogen compound metabolic process (GO:1901564), biosynthetic process (GO:0009058), organic substance biosynthetic process (GO:1901576), and others. The cellular component category mainly contained entries such as cellular anatomical entity (GO:0110165), intracellular (GO:0005622), organelle (GO:0043226), and so on, while the molecular function category included terms such as structural molecule activity (GO:0005198) and structural constituent of ribosome (GO:0003735) (Figure 3a, Supplement data 2: Table S3). The GO enrichment analysis of DEGs from comparing the SC and Org treatment groups (SC vs Org) showed that the biological process category includes terms such as biosynthetic process (GO:0009058), organic substance biosynthetic process (GO:1901576), and cellular biosynthetic process (GO:0044249). The cellular component category includes terms such as cellular anatomical entity (GO:0110165), intracellular (GO:0005622), and organelle (GO:0043226). In addition, the molecular function category mainly includes entries such as structural molecule activity (GO:0005198), structural constituent of ribosome (GO:0003735), and transcription regulator activity (GO:0140110) (Figure 3b, Supplement data 2: Table S5). We further divided the GO enrichment analysis of DEGs from SC vs NPK and SC vs Org treatment groups into up- and down-regulated expression (Supplement data 2: Table S4, Table S6). The results showed that the majority of the DEGs obtained from the two compared groups were concentrated in up-regulated expression regulation.

**Figure 4:**
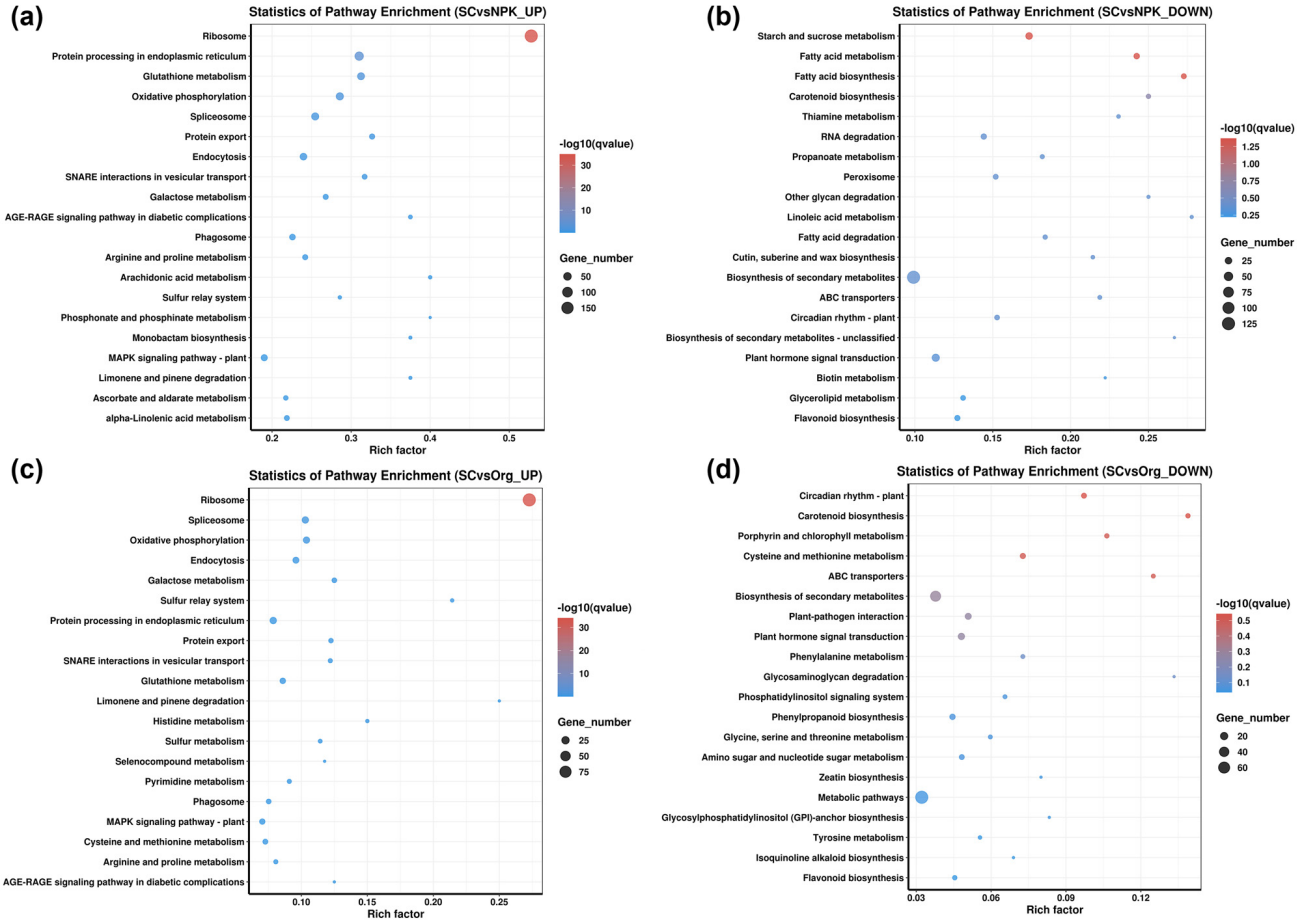
KEGG enrichment analysis of DEGs: (a) and (b) SC vs NPK; (c) and (d) SC vs Org.

DEGs were also employed to identify KEGG pathways. The comparison between the SC-treated group and the NPK-treated group revealed that up-regulated DEGs were primarily enriched in KEGG pathways such as ribosome (vvi03010), protein processing in endoplasmic reticulum (vvi04141), glutathione metabolism (vvi00480), oxidative phosphorylation (vvi00190), spliceosome (vvi03040), protein export (vvi03060), endocytosis (vvi04144), SNARE interactions in vesicular transport (vvi04130), and galactose metabolism (vvi00052) (Figure 4a, Supplement data 1: Table S3). In contrast, down-regulated DEGs were mainly enriched in pathways like fatty acid metabolism (vvi01212), fatty acid biosynthesis (vvi00061), and starch and sucrose metabolism (vvi00500) (Figure 4b, Supplement data 1: Table S3). The comparison between the SC-treated group and the Org-treated group revealed that up-regulated DEGs were mainly concentrated in KEGG pathways such as ribosome (vvi03010), spliceosome (vvi03040), oxidative phosphorylation (vvi00190), endocytosis (vvi04144), galactose metabolism (vvi00052), sulfur relay system (vvi04122), protein export (vvi03060), and protein processing in endoplasmic reticulum (vvi04141) (Figure 4c, Supplement data 1: Table S3). On the other hand, down-regulated DEGs were primarily enriched in pathways like carotenoid biosynthesis (vvi00906), circadian rhythm (vvi04712), and porphyrin and chlorophyll metabolism (vvi00860) (Figure 4d, Supplement data 1: Table S3).

### 3.4 Co-expression network

**Figure 5:**
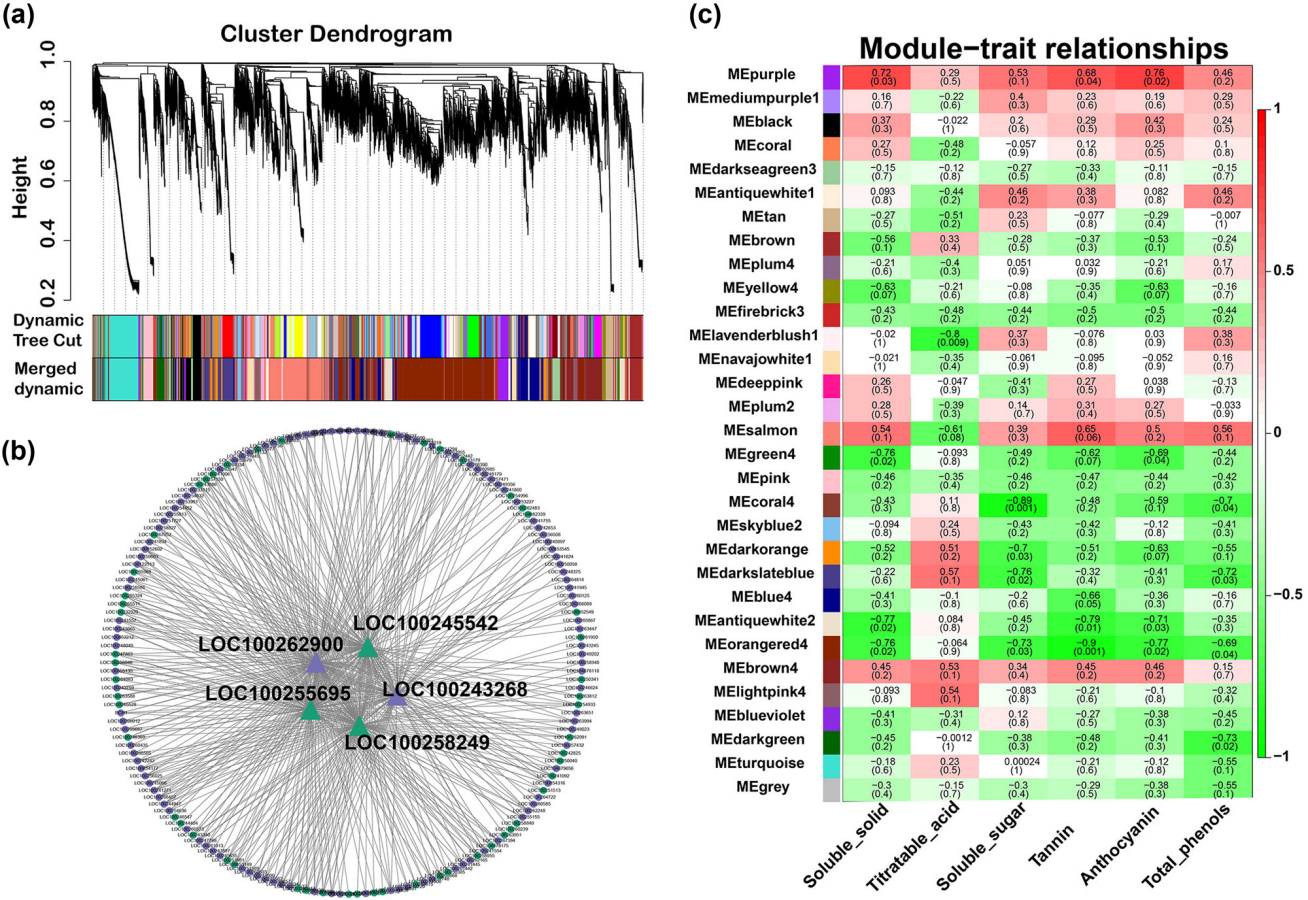
WGCNA identified gene networks. (a) Hierarchical clusters represent 31 distinct modules with co-expressed genes. Each leaflet in the tree represents an individual gene. (b) Pearson correlation-based module trait relationship. The color grids from green to red represent the *r* ^2^ value (−1 to 1). (c) Gene network of purple module. Hub genes are highlighted by triangles, and transcription factors are shown in green.

In order to explore the association between the regulatory network of grapevine root genes and berry phenotypes (soluble solid, titratable acid, soluble sugar, tannin, anthocyanin, and total phenols), WGCNA analysis was performed to globally identify highly correlated hub genes within highly connected gene networks based on the complete set of transcripts. Thirty-one modules were identified by applying the DynamicTreeCut function, and each module was visually distinguished by a series of color schemes (Figure 5a). By conducting Pearson correlation coefficient analysis, co-expression modules were identified for each grape fruit quality trait. The analysis results showed that the purple module, which contained 599 genes, had the highest correlation with soluble solids, soluble sugars, tannins, and anthocyanins (Figure 5b). Furthermore, we investigated key genes in the purple module. Through gene network construction, we obtained five hub genes, encoding Josephin-like protein (JP), 60S ribosomal protein L18a (60S), uncharacterized protein LOC100262900, and two transcription factors, namely, ethylene-responsive transcription factor (ERF) and splicing factor 3B subunit 6 (SF3B) (Figure 5c).

### 3.5 qRT-PCR analysis of anthocyanin synthesis genes

Considering that the application of SC resulted in higher anthocyanin content in grape berries compared to the two fertilization treatments, and that WGCNA analysis revealed a module of genes highly correlated with anthocyanin levels, we selected and validated the expression levels of five genes related to anthocyanin synthesis (three UDP-glucuronosyltransferases, glutathione-*S*-transferase, and anthocyanin acyltransferase) based on previous study [2]. In addition, we selected three genes for validation of their root transcription levels among the five hub genes identified in the gene network.

**Figure 6:**
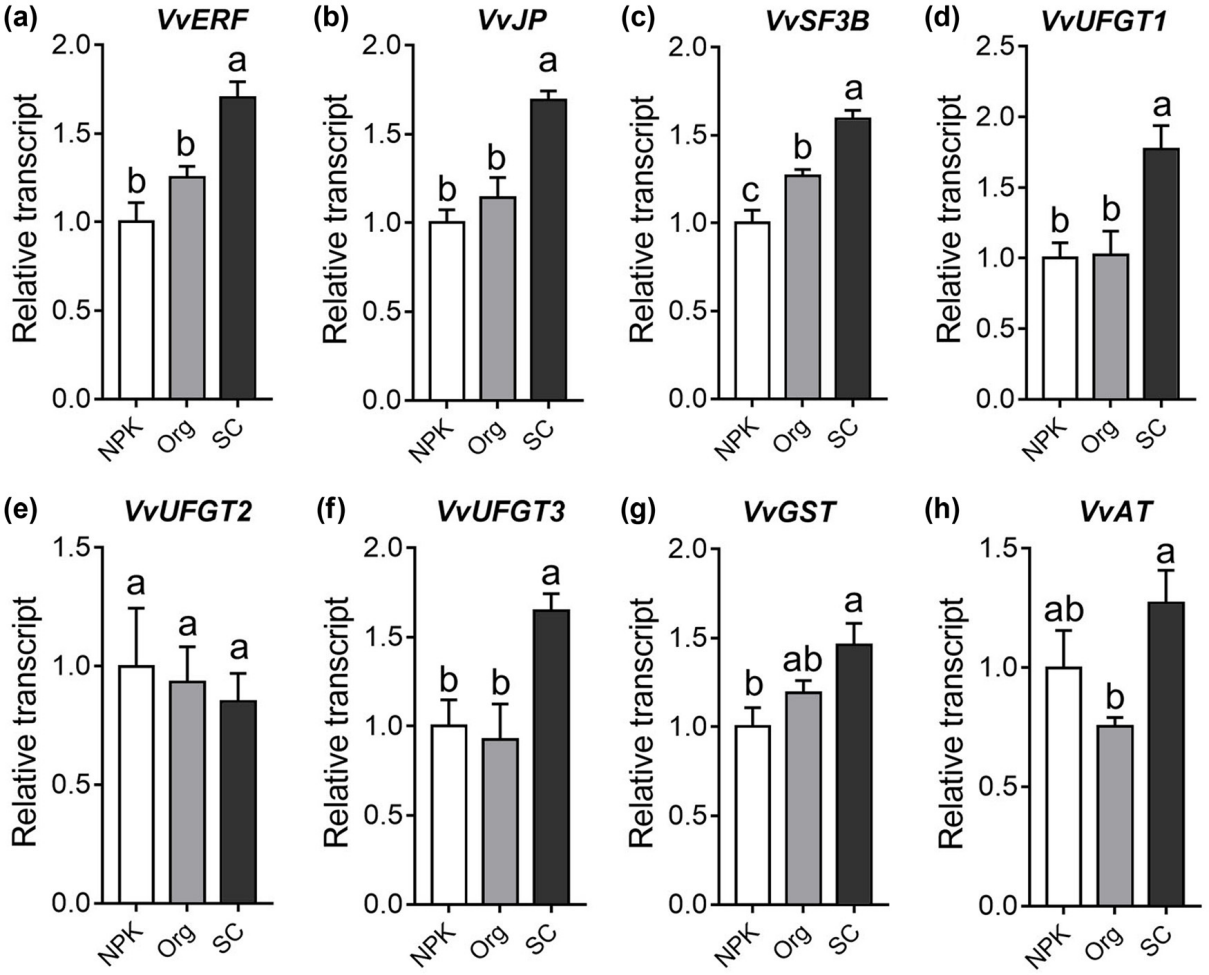
Expression levels of five genes involved in anthocyanin biosynthesis and three hub genes were validated by qRT-PCR. Three biological replicates are used for qRT-PCR validation. The averages (±SE) following different letters indicate significant difference at *p* value <0.05 using analysis of variance with the Tukey–Kramer test.

The qRT-PCR results showed that the expression levels of the three hub genes, *VvERF*, *VvJP*, and *VvSF3B*, were significantly higher in the SC-treated group than in the NPK- and Org-treated groups (Figure 6a–c). In addition, among the five genes related to anthocyanin synthesis, the transcript levels of *VvUFGT1* and *VvUFGT3* in the roots of grapevines treated with SC were significantly higher than those in the other two fertilizer-treated groups (Figure 6e and f). Moreover, the expression level of *VvGST* in the roots of the SC treatment group was significantly higher than that in the NPK treatment group (Figure 6g). The expression level of *VvAT* in the SC-treated group was significantly higher than that in the Org-treated group (Figure 6h). The transcript level of another UDP-glucuronosyltransferase gene, *VvUFGT2*, did not show significant differences between the SC-treated group and the other two treatment groups (Figure 6e).

## 4 Discussion

With increasing planting years, soil quality in wine grape vineyards deteriorates, particularly in terms of physical structure, chemical composition, and microbial diversity [25,26]. Failure to address these issues promptly reduces production capacity and lowers wine grape quality [27]. Therefore, rational soil management measures are urgently needed to improve soil quality and provide a favorable growth environment for grapevines. This study investigates the effects of three soil management measures on grapevine root system and fruit quality, with a focus on the advantages of SC compared to other two conventional fertilizers.

Soluble solids, titratable acidity, soluble sugars, tannins, anthocyanins, and total phenolics are considered important indicators of grape and wine quality [28], and fertilizers can have a significant impact on these quality indicators [2,8]. In this study, a SC was applied [29], which was formed by simple processing of lignite and contained approximately 38.5% humic acid. Grapes grown in soil treated with the SC had a higher proportion of soluble solids and soluble sugars compared to those grown in soil treated with inorganic and organic fertilizers, and their content of tannins, anthocyanins, and total phenolics also significantly increased (Figure 1). SCs can improve the physical and chemical properties of soil, and the humic acid they contain is known to stimulate plant growth and enhance nutrient uptake from the soil [30–32]. This can benefit root nutrient absorption and root system development, ultimately affecting fruit nutrient accumulation [33]. Under drought conditions, the application of humic acid at a concentration of 100–200 mg/L significantly enhanced the yield, dry weight, and root–shoot ratio of foxtail millet, while increasing the concentration of essential mineral elements such as P, Fe, Cu, Zn, and Mg in grains [34]. Humic acid has cytokinin, auxin, gibberellin-like activities, and contains 3-indoleacetic acid, which promote root growth and increase nutrient and water absorption surface area [35–37]. Thus, the use of humic acid as a foliar and soil application has been found to improve the growth, yield, and quality of coffee [37]. Moreover, humic acid can regulate soil microbial community, physical and chemical properties, and secondary metabolites in the bayberry rhizosphere, providing a novel strategy for managing bayberry decline disease [38]. Similarly, applying soil amendment in continuously cropped soil could lead to changes in the gene expression patterns in strawberry plant roots, such as an overall increase in the expression of nutrient transport genes and a decrease in the expression of defense response genes [39–41]. In this study, compared to the two fertilizer treatments, SC exerted distinct effects on the transcription levels of certain genes in grapevine roots (Figure 2). Among the up- and down-regulated DEGs, the most enriched GO terms showed that many up-regulated DEGs were associated with the biosynthesis of substances (Figure 3). KEGG pathway analysis revealed that SC promoted protein synthesis (Figure 4).

Anthocyanins are water-soluble flavonoids that contribute to the colors of plants. Key enzymes in the anthocyanin biosynthetic pathway include AT, UFGT, and GST. Potassium fertilizer application significantly increases the transcript levels of these enzymes in grape berries [2,42,43]. Similarly, in this study, treatment with SC significantly increased the transcript levels of UFGT1, UFGT3, GST, and AT in grapevine roots, potentially promoting potassium uptake and enhancing anthocyanin accumulation in grape berries [2]. Gene network analysis identified hub genes, including ERF, JP, and SF3B, which were significantly up-regulated in the SC treatment [2]. These genes play roles in regulating plant stress resistance and mRNA splicing [44–50]. Soil amendments can alleviate soil environmental stress, allowing plants to redistribute defense resources to development [39]. The utilization of SC may have impacted the microbial communities in the soil, leading to the activation of plant root genes responsible for stress resistance [51]. Further investigation is needed to understand the underlying molecular mechanisms [2,39,44–51].

## 5 Conclusion

In this study, we compared the effects of three soil management practices on grapevines, demonstrating that SC significantly improved grape fruit quality and altered the gene expression patterns in the plants compared to inorganic and organic fertilizers. Specifically, the application of SC significantly increased the soluble solids, soluble sugars, tannins, anthocyanins, and total phenols in grape fruits. Through WGCNA analysis of the transcriptomic data from the root of Cabernet Sauvignon, a module highly correlated with features such as anthocyanin was identified, with five hub genes identified within. We further validated three hub genes and specifically investigated five genes related to anthocyanin synthesis. The results showed that the expression levels of the three hub genes and *VvUFGT1* and *VvUFGT3* were significantly increased in the roots treated with SC. These findings suggest that improvements in soil quality, particularly with the application of SC, can effectively enhance grape quality.

## Supplementary Material

Supplementary Table 1

Supplementary Table 2
