# Supplementary material for: Improving grape fruit quality through soil conditioner: Insights from RNA-seq analysis of Cabernet Sauvignon roots
Source: Open Life Sci. 2024 May 7;19(1):20220864. doi: 10.1515/biol-2022-0864 (PMC11087741; doi:10.1515/biol-2022-0864)
Supplement: Supplementary Table 1 [file biol-2022-0864-sm.pdf]

Supplementary material

Table S1: The compositions of different fertilization tactics

Treatments	Composition
Nitrogen-phosphorus-potassium (NPK)	343.5 kg/hm^2 Urea (N ≥ 46%); 166.5 kg/hm^2 P_2O_5 (P_2O_5 ≥ 64%); 318 kg/hm^2 K_2O (K_2O ≥ 50%)
Organic fertilizer (Org)	9 t/hm^2 (Organic matter content ≥ 35%, N + P_2O_5 + K_2O ≥ 13%)
Soil conditioner (SC)	3 t/hm^2 (humic acid ≥ 38.5%; C, 56.1%; H, 3.7%; N, 1.5%; O, 38%; S, 0.6%)

Table S2: Summary of sequencing data

Sample	Raw reads	Raw bases	Clean reads	Clean bases	Clean reads ratio (%)	Q30(%)	GC(%)	rRNA mapping reads	rRNA mapping ratio	High quality clean reads	Mapping reads	Mapping ratio (%)	All Gene Num	Known gene Num	Novel gene num
NPK1	58614942	8792241300	58118850	8712484330	99.15	91.12	46.98	36534	0.06	58082316	43938333	75.65	21956	21145 (81.85%)	811
NPK2	47918724	7187808600	47423914	7109286097	98.97	90.65	47.44	100966	0.21	47322948	35445215	74.90	21732	20953 (81.11%)	779
NPK3	46332350	6949852500	45878270	6877625742	99.02	90.83	46.50	23556	0.05	45854714	34656595	75.58	21579	20794 (80.49%)	785
Org1	49249208	7387381200	48685048	7298066310	98.85	90.24	46.84	24802	0.05	48660246	35610478	73.18	20912	20158 (78.03%)	754
Org2	48552102	7282815300	48119420	7213276396	99.11	90.82	46.92	107056	0.22	48012364	33633762	70.05	21818	21013 (81.34%)	805
Org3	62793464	9419019600	62253082	9332303687	99.14	90.93	47.29	91974	0.15	62161108	46117460	74.19	22371	21547 (83.41%)	824
SC1	58208050	8731207500	57612988	8635709412	98.98	88.97	46.92	69698	0.12	57543290	41140995	71.50	18652	18048 (69.86%)	604
SC2	50020000	7503000000	49492324	7418599824	98.95	90.33	47.32	114966	0.23	49377358	35044020	70.97	20928	20188 (78.15%)	740
SC3	63205656	9480848400	62634614	9389445891	99.10	90.74	48.11	357052	0.57	62277562	44075912	70.77	20002	19297 (74.70%)	705

Table S7: Overview of KEGG analysis of DEGs

	Tendency	KEGG pathway	ID	Input	Total	*P*-Value
SC vs. NPK	Up-regulated	Ribosome	vvi03010	180	341	9.30×10^{-38}
		Protein processing in endoplasmic reticulum	vvi04141	67	216	6.82×10^{-7}
		Glutathione metabolism	vvi00480	40	128	1.01×10^{-4}
		Oxidative phosphorylation	vvi00190	44	154	2.52×10^{-4}
		Spliceosome	vvi03040	42	165	2.31×10^{-3}
		Protein export	vvi03060	16	49	8.35×10^{-3}
		Endocytosis	vvi04144	35	146	1.09×10^{-2}
		SNARE interactions in vesicular transport	vvi04130	13	41	1.98×10^{-2}
		Galactose metabolism	vvi00052	15	56	3.94×10^{-2}
	Down-regulated	Fatty acid metabolism	vvi01212	16	66	4.92×10^{-4}
		Fatty acid biosynthesis	vvi00061	12	44	1.03×10^{-3}
		Starch and sucrose metabolism	vvi00500	26	150	1.10×10^{-3}
		Carotenoid biosynthesis	vvi00906	9	36	6.89×10^{-3}
		ABC transporters	vvi02010	7	32	2.87×10^{-2}
		Linoleic acid metabolism	vvi00591	5	18	2.90×10^{-2}
		Fatty acid degradation	vvi00071	9	49	3.37×10^{-2}
		Thiamine metabolism	vvi00730	6	26	3.43×10^{-2}
		Biosynthesis of secondary metabolites	vvi01110	126	1271	3.79×10^{-2}
		Other glycan degradation	vvi00511	5	20	4.04×10^{-2}
		RNA degradation	vvi03018	15	104	4.22×10^{-2}
		Cutin, suberine and wax biosynthesis	vvi00073	6	28	4.47×10^{-2}
		Propanoate metabolism	vvi00640	8	44	4.59×10^{-2}
		Peroxisome	vvi04146	12	79	4.85×10^{-2}
SC vs. Org	Up-regulated	Ribosome	vvi03010	93	341	8.65×10^{-37}
		Spliceosome	vvi03040	17	165	5.54×10^{-3}
		Oxidative phosphorylation	vvi00190	16	154	6.52×10^{-3}
		Endocytosis	vvi04144	14	146	1.90×10^{-2}
		Galactose metabolism	vvi00052	7	56	2.81×10^{-2}
		Sulfur relay system	vvi04122	3	14	4.33×10^{-2}
		Protein export	vvi03060	6	49	4.39×10^{-2}
		Protein processing in endoplasmic reticulum	vvi04141	17	216	4.93×10^{-2}
	Down-regulated	Carotenoid biosynthesis	vvi00906	5	36	5.50×10^{-3}
		Circadian rhythm - plant	vvi04712	7	72	6.59×10^{-3}
		Porphyrin and chlorophyll metabolism	vvi00860	5	47	1.49×10^{-2}
		ABC transporters	vvi02010	4	32	1.76×10^{-2}
		Cysteine and methionine metabolism	vvi00270	8	110	1.79×10^{-2}
		Biosynthesis of secondary metabolites	vvi01110	48	1271	3.49×10^{-2}
		Plant-pathogen interaction	vvi04626	12	236	4.69×10^{-2}
		Plant hormone signal transduction	vvi04075	14	291	4.86×10^{-2}

Table S8: Gene primers for qRT-PCR

Gene number	Reference gene number in NCBI	Forward primer (5′–3′)	Reverse primer (5′–3′)
Vvactin	GU585869	CTTGCATCCCTCAGCACCTT	TCCTGTGGACAATGGATGGA
VvERF	XM_019226746	GCACAGAACAAAGGTCGGTT	ATCTCAGCCACCCACTTACC
VvJP	XM_019220434	GCTGAATGCCATAGCCGAAA	TACGGCGATCATGCCAAATC
VvSF3B	XM_010653232	AAACCCAAACCGGAAACCAC	ACGTAGAGGACTCGGTTCAC
VvUFGT1	XM_002285067	CTCGATGTGGAGAAGACAGC	CCTGAGACCTCGTCAATTCG
VvUFGT2	XM_002268053	GGGCGTGTCACATATTGTCT	GGGGAATTGGGAATGCTAGG
VvUFGT3	XM_002285734	CCAAACTCAACGACAGAGGT	ACTGCACATAGGCCATGAAG
VvGST	XM_003634701	GCTTCTTGAGATGAACCCGA	GGTATGGGTCACTAGGCAAC
VvAT	XM_010649854	GATGGATTCACTGCTGCTCA	TGGCAAGAAAGAGGCCAAAT
